# Skull Base Osteomyelitis: A Diagnostic Dilemma

**DOI:** 10.7759/cureus.17867

**Published:** 2021-09-10

**Authors:** Munira Ally, Hadyn Kankam, Abdul Qureshi, Arcot Maheshwar

**Affiliations:** 1 Otolaryngology, Colchester Hospital, Essex, GBR

**Keywords:** diagnosis, regression, necrotising otitis externa, malignancy, skull base osteomyelitis

## Abstract

Skull base osteomyelitis is a rare but potentially fatal condition. It is often characterised by a series of non-specific clinical and radiological signs, making it difficult to distinguish from a malignant lesion. We present the case of an immunocompetent elderly gentleman with multiple cranial nerve palsies and an unremarkable initial ear examination, diagnosed and treated for skull base osteomyelitis, masquerading as malignancy. This initially regressed without antibiotic therapy. This case emphasises the importance of clinicians having a high degree of diagnostic suspicion in order to initiate prompt treatment, thereby improving patient prognosis.

## Introduction

Skull base osteomyelitis (SBO) describes a rare and potentially fatal bone infection, with loss of cranial floor structural integrity [1,2]. It predominantly affects immunocompromised, diabetic, and elderly patients [3] and can be classified as typical or atypical SBO. Non-resolving otitis externa, refractory to antimicrobial agents, often precedes typical SBO, with the inflammatory process usually restricted to the temporal bone. However, atypical SBO affects the sphenoid and occipital bones (particularly the clivus) [4,5]. The presentation of SBO often varies, from severe headaches to lower cranial nerve palsies. Furthermore, its radiological appearance may mirror that of an infiltrative lesion such as malignancy [2]. Therefore, the combination of non-specific clinical and radiological signs present a diagnostic challenge, which may delay the initiation of appropriate treatment.

We describe an unusual presentation of SBO, masquerading as a neoplastic lesion, in an immunocompetent patient that partially regressed despite no medical intervention.

## Case presentation

A 78-year-old gentleman, with no previous otorhinolaryngological history, reported a four-week history of progressive dysphagia, dysphonia, and weight loss, following resolution of an ear infection treated with two courses of antibiotics. On examination, oral cavity, oropharynx, and external ears were unremarkable, with normal observations. He demonstrated weakness of right spinal accessory and winging of the scapula but no neck lumps. Flexible nasolaryngoscopy revealed nasal polyps and right vocal cord paralysis with dysphonia.

**Figure 1:**
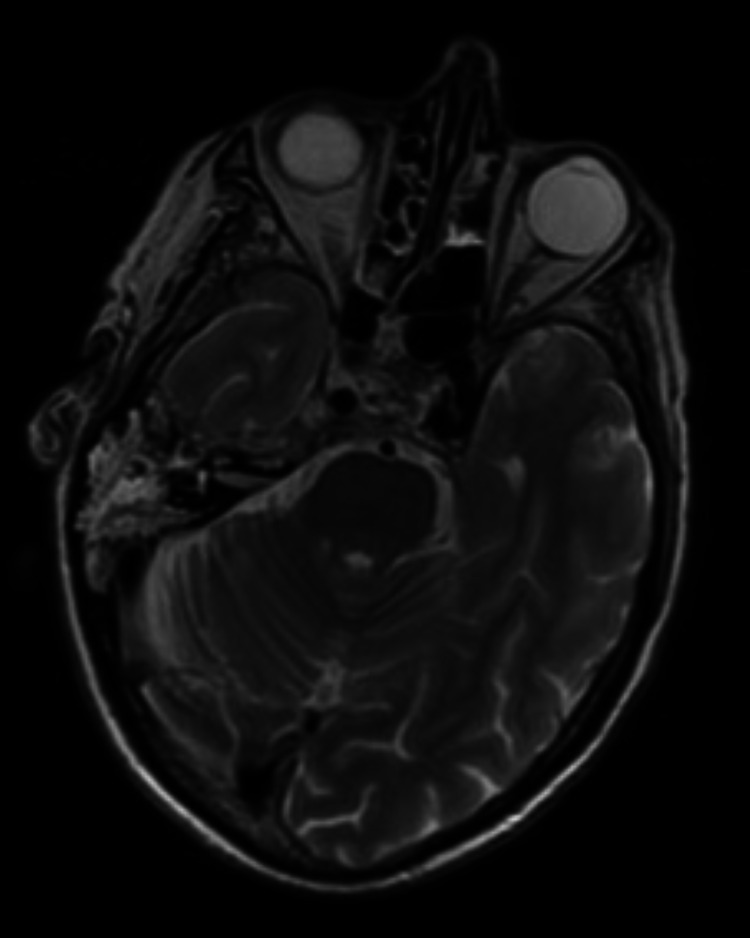
Initial MRI brain and neck of patient – T2-weighted.

Blood tests demonstrated a white cell count of 9x10^9/L (4x10^9/L - 11x10^9/L) and C-reactive protein (CRP) of 18mg/L (<4mg/L). A CT neck, chest and abdomen scan revealed circumferential thickening of the distal oesophagus, with oesophageal malignancy excluded on subsequent gastroscopy and biopsy. MRI of the brain and neck demonstrated abnormal thickening and enhancement in the right posterolateral nasopharyngeal mucosa with infiltrative pathology involving the right para-pharyngeal space, longus colli, carotid space, jugular foramen, right skull base, and clivus (Figure 1). Following this scan, he reported a deterioration in his hearing, with examination confirming a right middle ear effusion and mixed hearing loss. Positron emission tomography (PET)/CT showed a fluorodeoxyglucose 18F (FDG)-avid right nasopharyngeal lesion with probable erosion of the skull base. Subsequently, a biopsy of the postnasal space was performed and the right grommet was inserted. There was scanty serous fluid on myringotomy and no obvious postnasal space mass. Histology illustrated reactive lymphoid hyperplasia but no apparent carcinoma.

**Figure 2:**
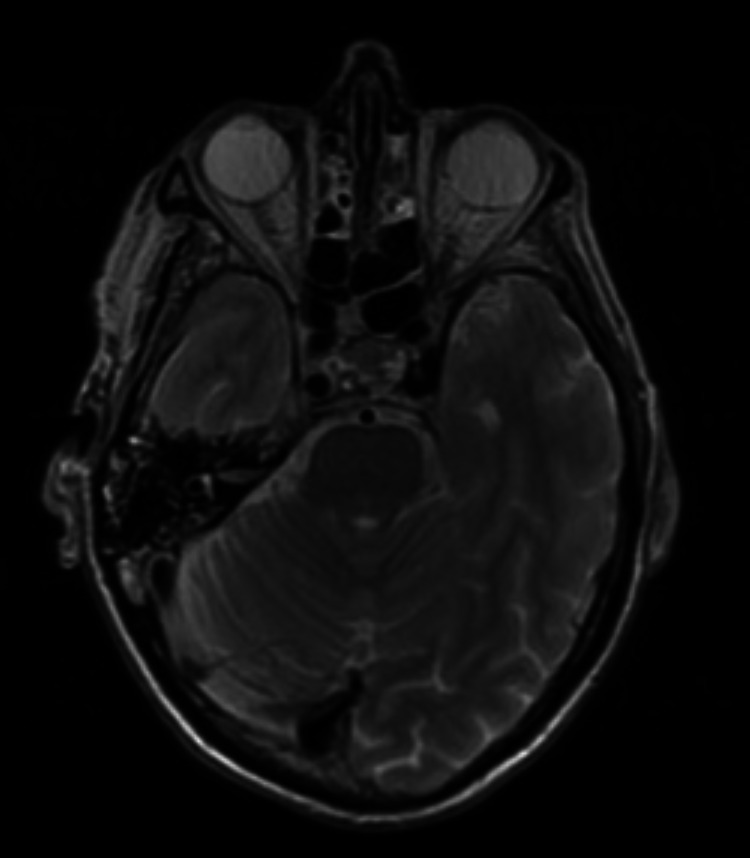
Repeat MRI brain and neck, three months after initial scan – T2-weighted.

The case was referred to the skull base multidisciplinary team (MDT) meeting and a CT of the skull base and sinuses was performed to ascertain the feasibility of endoscopic biopsy via the sphenoid sinus. This demonstrated significant regression of the right-sided skull base abnormality despite no medical treatment, three months after the initial CT scan. Repeat MRI illustrated consistent findings (Figure 2), favouring an infective cause for the disease process. Following discussion with the local microbiology team, the patient commenced intravenous ciprofloxacin and daptomycin therapy for SBO and received speech and language therapy. 

Upon completion of a six-week antibiotic course, his dysphagia had improved but dysphonia persisted. The patient was offered an injection of the right vocal cord; however, he opted for conservative treatment due to the COVID-19 pandemic and his wish to reduce his chance of contracting the virus by reducing time spent in public settings. We continue to review him as an outpatient.

## Discussion

A rare complication of otologic or rhinosinugenic infection, SBO is a potentially fatal clinical entity [2]. Malignant otitis externa secondary to *Pseudomonas aeruginosa* infection is implicated as the primary cause of typical SBO [6]. Atypical SBO affects the sphenoid bone, occipital bone, and clivus [4,5]. Distinction between subtypes may be challenging due to occult or partially treated infection prior to diagnosis [2]. Indeed, our patient had no clinical signs of infection at presentation but a preceding history of right ear infection, which may have been the initial focus.

Patients with SBO often present with a myriad of non-specific symptoms such as unremitting headaches with progression to cranial neuropathies and meningitis. Subtemporal spread of infection at the stylomastoid foramen can result in cranial nerve (CN) involvement, with CN VII being most commonly affected. Jugular foramen involvement may affect CN IX-XI as demonstrated in our case with the patient displaying dysphagia, unilateral vocal cord paralysis, and shoulder weakness. Spread to the petrous apex may result in CN V-VI palsy [1]. Malignancy is the commonest cause of multiple cranial neuropathies and, therefore, an important differential. Furthermore, involvement of nasopharyngeal soft tissue may cause eustachian tube obstruction and a middle ear effusion [2]; a common occurrence in nasopharyngeal malignancy. Thus, the cause of right middle ear effusion in this case with associated conductive deafness could have been attributed to either pathology. 

Blood tests generally demonstrate elevated acute phase reactants, often not seen in malignancy alone, thereby aiding the diagnostic process and guiding treatment response. Our patient did not have markedly raised inflammatory markers at presentation.

Radiological investigation often requires a multimodal approach. CT is frequently the initial investigation of choice and can demonstrate destructive bony erosion. However, this is not consistently present in biopsy-proven cranial osteomyelitis [7]. Thus, soft tissue inflammation may be the limit of findings in the early stages of the disease. MRI is superior in soft tissue discrimination and for evaluating intracranial complications associated with SBO [8] and, thus, is the preferred modality for follow up. Furthermore, radiological findings on CT may persist despite the completion of treatment [8]. Fluorodeoxyglucose (FDG)-PET/CT is relatively non-specific as FDG accumulates in tissue with high glucose demand, including infected and neoplastic lesions. 

Management of SBO requires an MDT approach to eradicate foci of infection and attenuate potential exacerbating factors. An extended course (up to six months) of antibiotics and/or antifungals, guided by culture and sensitivity analyses, is typically the mainstay of treatment. Clinicians should also seek to optimise management of patient co-morbidities including diabetes and immunodeficiency conditions where present [6]. Surgical intervention is typically reserved for cases where debridement or drainage of an abscess is required [1]. In selected cases, hyperbaric oxygen is efficacious in promoting local tissue oxygenation and thus the host’s immune response, whilst also demonstrating direct toxicity to microorganisms by oxidation [1]. Symptoms may take several months to completely resolve, but early therapeutic intervention is associated with improved prognoses [1].

## Conclusions

Due to similarities in the presentation, clinical, and radiological findings, distinguishing SBO from a neoplastic lesion can be challenging. Diagnosis is primarily guided by multimodal radiological, biochemical, and histopathological investigations. Management requires a multidisciplinary team approach with medical and occasionally surgical intervention required.
